# CASPER: Covert Channel Using Internal Speakers

**DOI:** 10.3390/s23062970

**Published:** 2023-03-09

**Authors:** Hyeongjun Choi, Ji Hyuk Jung, Ji Won Yoon

**Affiliations:** School of Cyber Security, Korea University, Seoul 02841, Republic of Korea; tiger8135@korea.ac.kr (H.C.); graycat@korea.ac.kr (J.H.J.)

**Keywords:** covert channel, high frequency sound, internal speaker

## Abstract

In recent years, researchers have studied various methods for transferring data in a network-separated environment, and the most representative method is the use of inaudible frequency signals like ultrasonic waves. This method has the advantage of being able to transfer data without other people noticing, but it has the disadvantage that speakers must exist. In a laboratory or company, external speakers may not be attached to each computer. Therefore, this paper presents a new covert channel attack that transfers data using internal speakers on the computer’s motherboard. The internal speaker can also produce a sound of the desired frequency, and, therefore, data can be transferred using high frequency sounds. We encode data into Morse code or binary code and transfer it. Then we record it using a smartphone. At this time, the location of the smartphone can be any distance within 1.5 m when the length per bit is longer than 50 ms, such as on the computer body or on the desk. Data are obtained by analyzing the recorded file. Our results show that data is transferred from a network-separated computer using an internal speaker with 20 bits/s in maximum.

## 1. Introduction

In computer security, a covert channel is a communication channel that transfers information through unknown channels. Therefore, this can be a means of communication that safely conveys information and at the same time can be a means of secretly transferring information. This technology is used when data is transferred in a network-separated environment where the organizations dealing with the security use. Computers connected to the internal network are not connected to the Internet and are blocked from connecting to the outside world. Since they believe that this place is safe to store their information assets, they normally save their crucial data. An adversary extracting the data from these computers could cause serious damage. To prevent this, many researchers study how to transfer information from a network-separated computer. In particular, research that transfers data by ultrasonic waves is likely to be used in actual attacks because it transfers data without being heard by others. However, it was impossible to generate ultrasound without an external speaker. Therefore, many companies where security is important restrict the use of external speakers.

Computers that do not have external speakers usually have internal speakers attached to the computer’s motherboard. This is because internal speakers are generally used to inform users what kind of error it is when an error occurs when booting a computer. Therefore, we propose a covert channel attack technique that uses internal speakers to transfer information. The attack scenario assumes that the computer is infected with a malware that elevates the privilege and we gain root privileges. Therefore, since we have root permission, we can use the internal speaker, which is assumed to be installed.

We experiment with sending data using internal speakers. For a successful attack, data is sent with high-frequency sounds that humans cannot hear. In this case, it is difficult for people around us to recognize this. At this time, the data is encoded in the form of Morse code or binary code. We receive high-frequency sound transmitted through an internal speaker through a mobile phone and analyze it using an application within the mobile phone to decode the transmitted data. Similar to the actual attack environment, this experiment is conducted in a network-separated environment without the Internet. Experimental results are presented by calculating the transmission rate as a bit rate, and we also present countermeasures against our attack. This paper demonstrates that beep sound is also a communication channel to be considered as a covert channel from a security perspective. We make the following contributions.

- We propose a method of using internal speakers as a method of performing secret channel attacks in air-gapped networks. Accordingly, it is possible to include a user who does not use an external speaker in the event of a covert channel attack, thereby expanding the scope of the attack.
- We encode in Morse code when transferring alphabets and binary code when transferring other files such as images. This shows that our attack can transfer information in various formats. When receiving and decoding this, an application on a smartphone is used. These experiments show that our attacks can be utilized in a realistic environment.
- We propose a countermeasure to defend our method. One is policy way to eliminate the means of attack and the other one is physical way to detect our attack.

## 2. Related Work

In this paper, the definition of a covert channel refers to a channel through which information is delivered in a manner other than any known communication channel. This paper especially deals with the methodology to transfer data from air-gapped computer in an attacker’s point of view. Recent studies have mainly used electromagnetism, magnetism, optics, temperature, and sound as a method used as covert channel.

Radio is currently the mostly used communication channel. In order to use it as a covert channel, it must be possible to generate a specific electromagnetic wave on a computer without any external device. Air hopper [1] uses screen cables to generate these without an external device. USBee [2] generates electromagnetic using a USB data bus. BitJabber [3] also generates electromagnetic using DRAM bus.

A method of constructing a covert channel using magnetic field energy is also an area to be studied. In the latest study, a CPU core [4] or a magnetic head [5] of hard disk were used to generate magnetic field energy. For reception, a magnetic field sensor built in a smartphone was used.

There have also been many studies on covert channels using optical emanation. A method of transferring information using computer keyboard LEDs [6], hard driver indicator LEDs [7], and LEDs of routers and switches has been proposed [8]. A method using the blinking of an LCD screen has also been studied [9].

It was also studied that technology of transferring information using heat. BitWhisper [10] succeed in transferring information between two adjacent air-gapped computers without any thermal generator.

Sound was also used to transfer information. To generate sound on a computer, the computer can use a speaker. Therefore, in order to use sound in covert channel, an important premise is mainly focused on delivering information without people around recognizing it and receiving sound without any device (microphone). The issues were solved by using ultrasonic signals [11,12] and jack retasking [13].

## 3. Proposed Approach

In our attack scenario, it is assumed that an attacker infects malicious code to a victim computer with a Linux operating system and then controls all privileges. The way to insert malicious code into a computer from a network-separated computer is to insert malicious code into a software update file by using USB containing malicious code or attacking a software company. Many companies and laboratories restrict the use of external speakers for several reasons, such as security. Therefore, our experiment also assumes that the computer does not have speakers except for internal speakers. Therefore, we propose an attack that uses the computer motherboard of the internal speaker to extract information. Usually, internal speakers are used to tell users if there are any errors during computer booting and whether they booted normally. Therefore, most computers without external speakers also have internal speakers.

The attacker uses near ultrasound (17 kHz to 20 kHz) that adults cannot hear well to transfer confidential information from air-gapped computers to the outside through internal speakers. The attacker can control the internal speaker using the ioctl request KIOCSOUND in Linux, the victim’s computer operating system. The clock tick rate is 1,193,180 on Linux. Thus, a frequency may be designated in the form of 1,193,180÷frequency as a factor to produce a sound.

**Figure 1:**
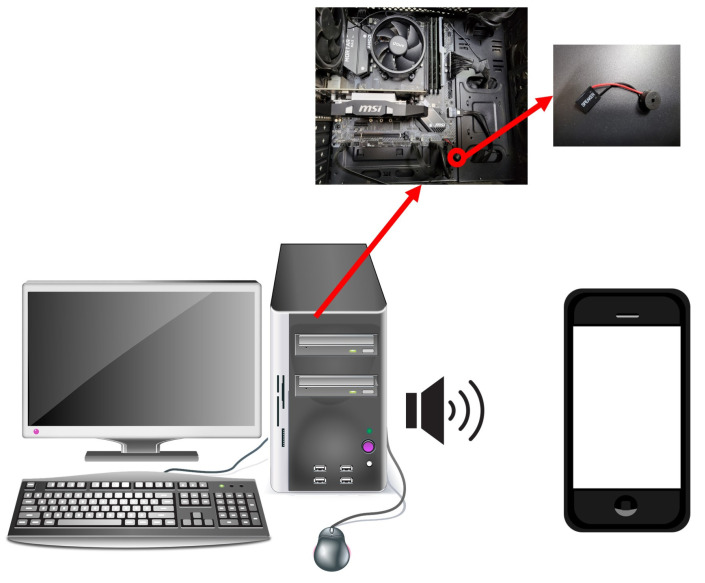
Diagram that shows the overall process of the algorithm.

Receivers can be used with all recording devices, but smartphones will be the most convenient of them. It is because other recording devices only have the ability to record ultrasound from internal speakers, there is a hassle of having to transfer them to another computer for decoding. However, in the case of smartphones, secret information can be obtained by decoding immediately after recording using an application. The overall picture of our covert channel attack is shown in Figure 1.

### 3.1. Encoding Method for Sender

Morse code requires a distinction between a dot (·) and a dash (-), and binary code requires a distinction between 1 and 0. In order to separate this in our scenario, we use the frequency method. There are usually Amplitude Modulation (AM) and Frequency Modulation (FM) methods used to encode and send data. AM works by varying the intensity of the signal to be transferred with respect to information to be sent. FM changes only the frequency without changing the intensity of the signal. The AM method cannot be used because the strength of the signal cannot be adjusted using the internal speakers we use. However, we can use the FM method because we can change frequencies using internal speakers. By using the FM method, data can be classified by sending low frequencies for a dot or 0 and high frequencies for a dash or 1. As a result of our experiment, it was confirmed that the frequency error reached up to 300 Hz. Therefore, it is preferable that the difference between the low frequency and the high frequency is 600 Hz or more in order to easily distinguish the frequencies. So, in this paper we used 1000 Hz.

It is recommended that the encoding varies depending on the type or size of data to be sent. One of the well known methods to encode data is Morse code. It transfers English alphabets or numbers using a dot and a dash. Morse code uses three times the length of a dot as a dash. In addition, the separation between the alphabets is three times the length of the dot. When distinguishing signals by frequency differences, only alphabets need to be distinguished. In that case, we use three times the length of the signal we sent.

The other way to encode data is to use binary code. This allows us to transfer simple data such as a secret key and files. Using this method, it is not necessary to distinguish alphabets from each other like in Morse code, but it is difficult to confirm that the data is properly delivered. Therefore, it is better to send data according to certain rules than to just send it raw. We used a method of displaying the start time using a frequency lower than 1000 Hz. We define one bit that we send as a start bit. In addition, even parity bit is used to verify integrity. There are many integrity verification methods, but in this experiment, we use an even parity bit because we transfer short data and the probability of errors is low. End bit can also be used to inform the end of the data. We also define the end bit as using twice the length of the start bit to set 1000 Hz lower.

### 3.2. Decoding Method for Receiver

An attacker records the transferred data with a smartphone. The attacker needs to decode the recorded data to obtain the original data because the data is encoded and transferred. The recorded signal must be interpreted as binary data to decode the transferred data. If the transferred signal length is short, the attacker can convert it into a spectrogram and check it with his eyes. However, if signal length is long, it is difficult to convert. Therefore, this method is not used in this paper. We propose to automate the decoding process of the transferred signal according to the format of the data. First, the frequency is extracted from the recorded file. In addition, the extracted frequencies are decoded based on the number of specific frequency bands. A specific frequency band is determined by considering an error of about 300 Hz in the transmitted frequency band. Frequency is extracted every 25 ms from the recorded file. Therefore, if an attacker sends a signal to a length of 100 ms, the four extracted frequencies form one signal.

## 4. Experimental Results

In this section, we experiment with transferring data from computers in an network-separated environment to smartphones using beep sound waves. The Morse code and the binary code may be transferred to the smartphone through beep sound waves as a result of the experiment. In the experiment, sound waves are generated through an internal speaker without an external speaker device, and signals are generated through this. We also calculate the bit error rate according to the distance the smartphone is located and the length of the beep per bit. Using this, we also propose the location of the best smartphone when using our attack.

### 4.1. Attack Preparation

We experiment in an environment of Ubuntu 20.04.1 64 bit. Ubuntu kernel version is 5.11.0-27-generic. However, our method allows data transmission regardless of computer specifications under our control. Our covert channel technology is available as long as the computer has an internal speaker. The computer does not have an external speaker and is not connected to the Internet. Recording the sound transferred by the internal speaker does not matter to any device. In our case, in order to create a situation similar to reality, we used mobile phones used in our daily lives without using professional equipment. We use Galaxy Z Filp3 5G (SM-F711N). We put a smartphone on the computer and recorded the sound. The distance between the internal speaker and the smartphone is about 20 cm. The basic recording application of the mobile phone we used in the experiment is set to record sounds with a sampling frequency of up to 20 kHz.

### 4.2. Overall Performance

**Figure 2:**
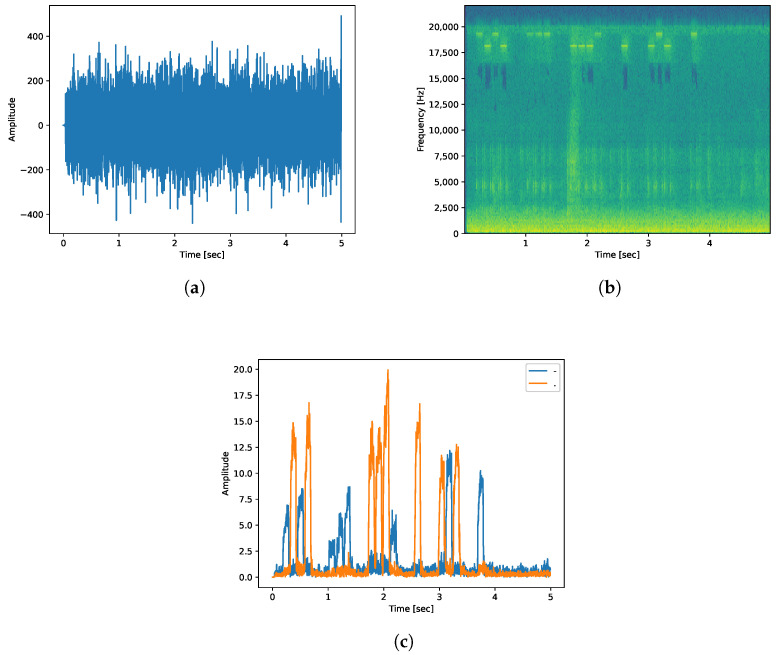
Time waveform, spectrogram, and frequency amplitude signals of sound encoding the word “covert” using beep sound recorded by a smartphone at a distance of 50 cm. These figures allow visual identification of data when transferring simple data. (**a**) Time waveform of sound transferring Morse code “covert” using beep sound. (**b**) Spectrogram of sound transferring Morse code “covert” using beep sound. (**c**) Frequency amplitude signals of sound transferring Morse code “covert” using beep sound.

In the experiment of encoding and transferring data with Morse code, the length per bit was set to 100 ms. We sent a dot at 18 kHz and a dash at 19 kHz. The spectrum and frequency amplitude signals that transfer the word “covert” in Morse code at a distance of 50 cm are shown in Figure 2. The spectrogram has parts that stand out as bright lines in the 18 kHz and 19 kHz parts. We can figure out the word “covert” using these bright lines. We can obtain the word “covert” by looking at the frequency amplitude and separating the dash and dot with blue and orange lines.

**Figure 3:**
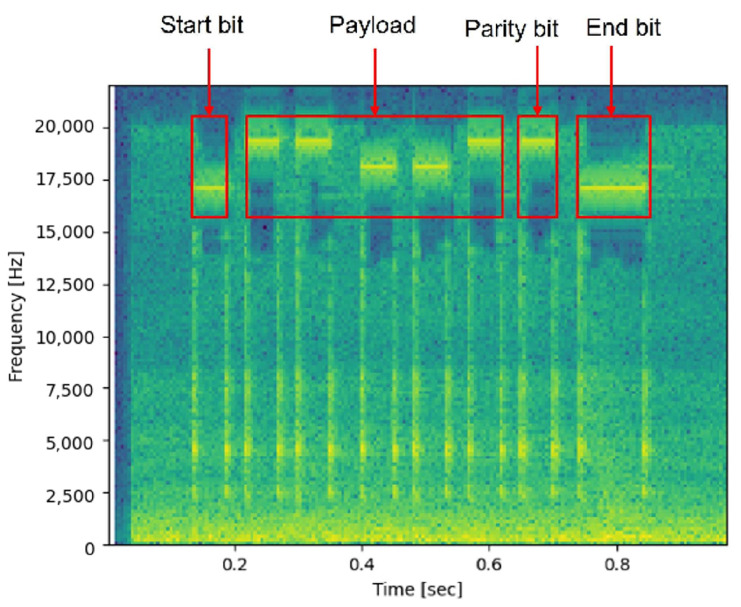
Spectrogram of sound transferring binary code “11001” using beep sound.

In the experiment of encoding data with binary code, the length per bit was set to 50 ms. We conducted an experiment transferring an arbitrarily determined binary code, 11001. We transferred the 50 ms long start bit at a frequency of 17 kHz. The 0 was transferred at a frequency of 18 kHz and 1 transferred sent at a frequency of 19 kHz. The next bit was an even parity bit. The even parity bit becomes 0 if the total number of 1-bits is even, and 1 if odd. In our experiment, the parity bit was 1. The last bit was the end bit. In this experiment, the end bit is the same frequency as the start bit at 17 kHz and the length is twice as long as 100 ms. The results of our experiment are shown in Figure 3.

### 4.3. Select Magic Numbers

In previous experiments, we arbitrarily set the length of one bit and the distance between the internal speaker and the smartphone. We experimented by varying the distance between computers and smartphones and the length of one bit. For this experiment, we introduce a new metric by bit error rate (BER).

(1) $$bit\;error\;rate=\frac{number\;of\;bit\;errors}{number\;of\;bits\;transferred}.$$

**Figure 4:**
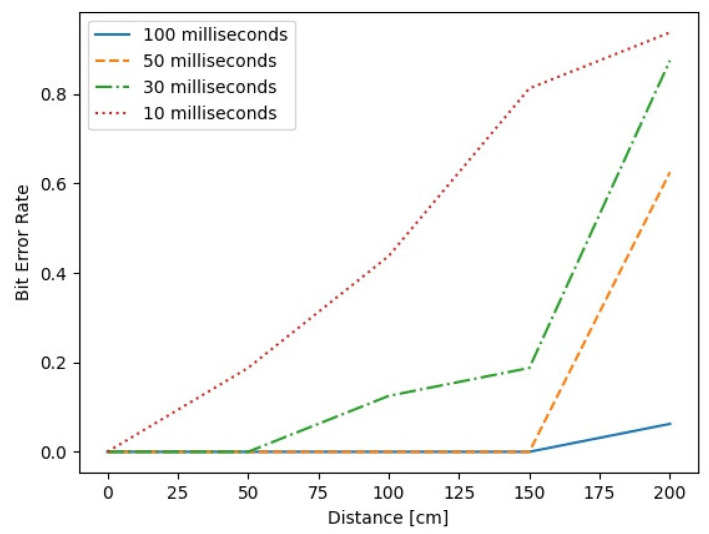
Bit error rate for distance and length per bit.

If the distance is close, it is decoded without error even if the length per bit is short. However, as the distance increases, the shorter the length of one bit, and the error bits increase. Therefore, we put a smartphone on a computer body about 20 cm away from the internal speaker and conducted an experiment, with a length of 100 ms or 50 ms per bit. As shown in Figure 4, our results show that if the length per bit is 100 ms, it is possible to transfer a bit at a distance of up to 1.5 m without error. This result shows that the maximum bit rate is 20 bits/s when the length per bit is 50 ms.

## 5. Discussion

Countermeasure can be a policy method. Usually, network separation is often used where security is considered important. Therefore, there will be internal security regulations. The simplest and most reliable way to prevent our scenario is to remove or selectively install internal speakers only if necessary. If there is no internal speaker, our method cannot transfer data. Therefore, it will be possible to prevent data transferred of the internal network by adding the presence or absence of internal speakers to the company’s internal security checklist. Another method is to install a device that detects abnormal signals in inaudible frequencies. Using such a device makes it difficult to use covert channel attacks using high frequencies that are difficult to hear. The other method is to use electronic filtering techniques. For example, a high-pass filter could be used to attenuate frequencies below a certain threshold, effectively blocking ultrasonic waves. However, the effectiveness of electronic filtering would depend on the quality of the filter design and the accuracy of the device’s microphone or other sensors.

Our method is slower in transferring data compared to other covert channel technologies using optical methods or electromagnetic methods because the speed of data transfer by sound is limited. Therefore, it is possible to think of a method of transmitting data simultaneously by varying the frequency band for a fast bit rate. However, this method is impossible. The method using an internal speaker is impossible to produce sound in one frequency band while making sound in another frequency band. When we tried it, the sound was transmitted only to the frequency band entered later.

## 6. Conclusions

In this paper, we present a new method of transferring data in a network-separated environment. Known techniques of using external speakers cannot be used in environments without external speakers. Therefore our method uses an internal speaker attached to the computer’s motherboard. We demonstrate how to produce inaudible frequency signals using internal speakers and how to transfer data using them. Our method shows that data is transferred from a computer to a smartphone at a distance of 1.5 m at up to 20 bits/s. Our results show that data can be transferred using near ultrasonic waves without external speakers. Through our research, it is possible for computers without external speakers to make sounds through internal speakers, and it is suggested to remind organizations of the possibility of data being transferred this way and to take measures to prevent these sounds.

## Data Availability

Not applicable.

## Abbreviations

The following abbreviations are used in this manuscript:

AM	Amplitude Modulation
FM	Frequency Modulation
BER	Bit Error Rate
